# Genome-Wide Identification of Destruxin A-Responsive Immunity-Related MicroRNAs in Diamondback Moth, *Plutella xylostella*

**DOI:** 10.3389/fimmu.2018.00185

**Published:** 2018-02-08

**Authors:** Muhammad Shakeel, Xiaoxia Xu, Jin Xu, Shuzhong Li, Jialin Yu, Xianqiang Zhou, Xiaojing Xu, Qiongbo Hu, Xiaoqiang Yu, Fengliang Jin

**Affiliations:** ^1^College of Agriculture, South China Agricultural University, Laboratory of Bio-Pesticide Creation and Application of Guangdong Province, Guangzhou, China; ^2^Beijing Genomic Institute, Shenzhen, China; ^3^School of Life Sciences, Institute of Insect Science and Technology, South China Normal University, Guangzhou, China

**Keywords:** microRNAs, destruxin A, immunity, innate, *Plutella xylostella*, differential expression analysis

## Abstract

*Plutella xylostella*, a global key pest, is one of the major lepidopteran pests of cruciferous vegetables owing to its strong ability of resistance development to a wide range of insecticides. Destruxin A, a mycotoxin of the entomopathogenic fungus, *Metarhizium anisopliae*, has broad-spectrum insecticidal effects and has been used as an alternative control strategy to reduce harmful effects of insecticides. However, microRNA (miRNA)-regulated reactions against destruxin A have not been elucidated yet. Therefore, here, to identify immunity-related miRNAs, we constructed four small RNA libraries from destruxin A-injected larvae of *P. xylostella* at three different time courses (2, 4, and 6 h) with a control, and sequenced by Illumina. Our results showed that totally 187 known and 44 novel miRNAs were identified in four libraries by bioinformatic analysis. Interestingly, among differentially expressed known miRNAs, some conserved miRNAs, such as miR-263, miR-279, miR-306, miR-2a, and miR-308, predicted to be involved in regulating immunity-related genes, were also identified. Worthy to mention, miR-306 and miR-279 were also listed as common abundantly expressed miRNA in all treatments. The Kyoto Encyclopedia of Genes and Genomes pathway analysis also indicated that differentially expressed miRNAs were involved in several immunity-related signaling pathways, including toll signaling pathway, IMD signaling pathway, JAK–STAT signaling pathway, and cell adhesion molecules signaling pathway. To the best of our knowledge, this is the first comprehensive report of destruxin A-responsive immunity-related miRNAs in *P. xylostella*. Our findings will improve in understanding the role of destruxin A-responsive miRNAs in the host immune system and would be useful to develop biological control strategies for controlling *P. xylostella*.

## Introduction

In nature, with the passage of time, a number of new mechanisms of host defenses have been developed in the arms race of host–pathogen. Animals face pathogen challenge with innate and adaptive immune system whereas, invertebrates, unlike mammals, do not have an adaptive immune system, but instead they rely on a sophisticated innate immune system for defense against invading microbes (1). The innate immune system of insects is comprised of two main components, cellular and humoral immune responses (2). The former relies majorly on the action of hemocytes in the phagocytosis of pathogens (3), while the latter refers to the process of melanization with phenoloxidases (4) and synthesis of immune effector molecules (5). Upon microorganism invasion, the immune system reactions are initiated by the recognition proteins, including peptidoglycan recognition proteins, β-1,3-glucan recognition proteins (βGRPs), galectins, C-type lectins, and scavenger receptors (6). The recognition step then leads to amplification of signals by serine proteases and triggers the activation of immune signaling pathways followed by induction of antimicrobial peptides to clear the infection (7, 8).

MicroRNAs (miRNAs), a class of small RNAs (sRNAs), are reported to play a pivotal role in the gene expression regulation at the level of posttranscription in many organisms. Since the discovery of the first miRNA in *Caenorhabditis elegans* (9), many miRNAs have been identified in plants, animals, and insects (10), and our knowledge of miRNA interaction with targets is persistently increasing and developing with the passage of time. In insects, miRNAs have been demonstrated to be key mediators in various physiological processes, including embryonic development (11), apoptosis (12), morphogenesis (13), and cell differentiation (14), and a recent report demonstrated that miRNAs may also play crucial roles in the regulation of innate immunity (15).

The entomopathogenic fungi, such as *Metarhizium anisopliae* and *Beauveria bassiana*, are considered as an environmentally friendly approach for the control of insect pests. During pathogenesis, these entomopathogenic fungi secrete virulence factors to accelerate the death of infected host (16). Destruxins, the virulence factors of fungi, have been reported to exhibit high toxicity to various insect species when ingested or injected (17–19) and inhibit V-type ATPase hydrolytic activity, prompt oxidative stress, and affects the Ca^2+^ channel in muscle cells (20–22). Additionally, destruxins play a pivotal role in the immune system of insects, such as *Drosophila melanogaster*; innate immune response was suppressed by destruxin A following the inhibition of antimicrobial peptides (16), whereas the immune system of *Bombyx mori* was induced in response to destruxin A (23). Although recently, the number of studies reporting immunity-related genes in *Plutella xylostella* is growing (24–27), thanks to the availability of *P. xylostella* genome, however, information regarding miRNA-regulated reactions in insect–pathogen interactions is still in its infancy and, to the best of our knowledge, there is no information available on miRNA-regulated reactions against the secondary metabolites of fungi, especially destruxin A.

Keeping in view the importance of miRNAs in insect–pathogen interaction, as exhibited by previous reports, we hypothesized that the network of miRNA-guided gene expression regulation might play a pivotal role in the control of innate immunity in destruxin A-infected *P. xylostella*. Furthermore, we also intended to find out that how *P. xylostella* miRNAs respond to destruxin A at different time courses of infection and whether the immune system of *P. xylostella* has developed novel miRNAs to combat the infection of destruxin A. To address these questions, a high-throughput sequencing Illumina and real-time quantitative PCR (RT-qPCR) were carried out to identify unique, novel, and immune-responsive miRNAs in *P. xylostella* infected with destruxin A at three different time courses (2, 4, and 6 h).

## Materials and Methods

### Rearing of Insects and Destruxin A Preparation

A susceptible *P. xylostella* strain was obtained from the Engineering Research Centre of Biological Control, Ministry of Education, South China Agricultural University, China and was kept insecticide-free for 10 generations. *P. xylostella* were maintained at 25 ± 1°C, 65% relative humidity, and a 14:10 h (light: dark) photoperiod. Destruxin A was extracted from *M. anisopliae* strain MaQ-10. The purity of destruxin A was evaluated by high-performance liquid chromatography. Finally, destruxin A was diluted in phosphate buffered saline (PBS, PH 7.4).

### Destruxin A Injection, sRNA Library Construction, and Sequencing

To inject destruxin A into the susceptible fourth instar larvae of *P. xylostella*, a stock solution of destruxin A (200 μg/mL) was prepared, and then 2 μL of that solution was injected to each larva. The control group was treated with PBS. A number of 30 larvae were collected from each treatment (2, 4, and 6 h post-injection) and control following instant freezing in liquid nitrogen. Trizol Total RNA Isolation Kit (Takara, Japan) was used to extract total RNA following manufacturer’s instructions. The concentrations of RNA were assessed using Nanodrop (Bio-Rad, USA) and its integrity was determined on Agilent 2100 Bioanalyzer (Agilent, USA).

Furthermore, RNAs were firstly ligated with 3′ adapter and after size fraction, ligated to 5′ adapter. The sRNA fractions were then used for reverse transcription following PCR. The final ligation PCR products, after purification, were sequenced using Illumina Genome Analyzer (San Diego, CA, USA) at the Beijing Genomics Institute (BGI, Shenzhen, China).

### Bioinformatics of Destruxin A-Responsive sRNA Sequences

To obtain clean reads from raw data reads having low-quality, 5′ primer contaminants, without 3′ primers and insert tag, and sequences fewer than 18 nucleotides (nt), were filtered out. To analyze the distribution, the final clean reads of the four libraries were mapped to *P. xylostella* genome (GCA_000330985.1) using Bowtie program (28). All the remaining clean sequences were annotated into different classes to remove rRNA, scRNA, snoRNA, snRNA, and tRNA using Rfam database. Finally, the unannotated clean sequences were used to predict novel miRNAs using the miRDeep2 software.

### Differential Expression Analysis of Destruxin A-Responsive miRNAs

The expression of miRNAs was compared between treatment and control to identify differentially expressed miRNAs. First, the expression of miRNA in the four libraries was normalized to transcripts per million. If the normalized expression of the miRNA was 0, it was modified to 0.01 to enable calculation. If the normalized expression of the miRNA was less than 1 in all libraries, it was ignored to compare for low expression. The normalization formula was

$$\text{Normalized expression}=\text{actual miRNA count/total count of clean reads}\times 10^{6}.$$

The normalized data were then used to calculate fold-change values and *P*-values, and a scatter plot of the fold-change values was generated. Fold-change was calculated as

$$\text{Fold-change}={\text{log}}_{2}\left(\text{treatment/control}\right).$$

The *P*-value was calculated by the following equation:

$$p\left(x|y\right)={\left(\frac{N_{2}}{N_{1}}\right)}^{y}\frac{\left(x+y\right)!}{x!y!{\left(1+\frac{N_{2}}{N_{1}}\right)}^{\left(x+y+1\right)}}\begin{array}{c} C\left(y\leq y_{\text{min}}|x\right)=\sum_{y=0}^{y\leq y_{\text{min}}}p\left(y|x\right)\\ D\left(y\geq y_{\text{max}}|x\right)=\sum_{y\geq y_{\text{max}}}^{\infty }p\left(y|x\right) \end{array},$$

where *x* represents sRNA total clean reads in the control, *y* represents total clean reads in the treatment, *N_1_* represents the normalized expression of a miRNA in library control, and *N_2_* represents the normalized expression of the same miRNA in library treatment. The corrected *P*-value corresponds to differential gene expression test using Bonferroni method (29).

### Genome-Wide Target Prediction for Destruxin A-Responsive miRNAs

To predict and analyze potential targets of differentially expressed miRNAs, three different software, including RNAhybrid (30), miRanda (31), and TargetScan (32) were used following the principles of target prediction as described previously elsewhere (33, 34). To increase the level of confidence and get more reliable results, we selected only those binding sites that were predicted by all three software.

### Gene Ontology (GO) Enrichment and Kyoto Encyclopedia of Genes and Genomes (KEGG) Pathway Analysis of Predicted Targets of Destruxin A-Response miRNAs

The genome database of *P. xylostella* was used as the background to determine GO terms enriched within the predicted targets dataset using hypergeometric test and a corrected *P*-value (≤0.05) as a threshold in order to find out significantly enriched terms. Finally, KEGG pathway enrichment analysis was performed to identify significantly enriched pathways within the predicted targets datasets compared with the genome database using hypergeometric test and a corrected *P*-value (≤0.05) as a threshold.

### Verification of Destruxin A-Responsive miRNAs by RT-qPCR

Real-time quantitative PCR is the method of choice for analyzing expression of genes and to confirm the results of RNA-Sequencing (35). The RT-qPCR analysis was conducted to ensure the expression levels of miRNAs displayed by Illumina sequencing results and 10 differentially expressed miRNAs were selected from the comparison of control vs. treatments. Total RNA was extracted from each sample as described earlier. The RNA sample (1 μg) was treated with DNaseI (Fermentas, Glen Burnie, MD, USA) following manufacturer’s protocol and then complementary DNA was synthesized using M-MLV reverse transcriptase (Promega, USA). The RT-qPCR was carried out on a Bio-Rad iQ2 optical system (Bio-Rad) using SsoFast EvaGreen Supermix (Bio-Rad, Hercules, CA, USA) following the manufacturer’s guidelines. The amplification cycling parameters were: 95°C for 30 s, 40 cycles of 95°C for 5 s, and 55°C for 10 s with a dissociation curve generated from 65–95°C to ensure the purity of PCR products (36). The U6 snRNA was used as an internal control for normalization and the relative expression of genes was calculated using the 2^−ΔΔCT^ method (37). Each experiment was replicated in triplicate.

### Statistical Analysis

Statistical analyses of the present study were performed using SPSS software (version 22.0; IBM Corp., Armonk, NY, USA). The differences between treatments were compared using Student’s *t*-test or one-way analysis of variance followed by Tukey’s test for multiple comparisons at the following significance levels: lowercase letters at *P* < 0.05 and capital case letters at *P* < 0.01. All results are expressed as means ± SEM.

## Results

### Construction of sRNA Libraries

**Figure 1:**
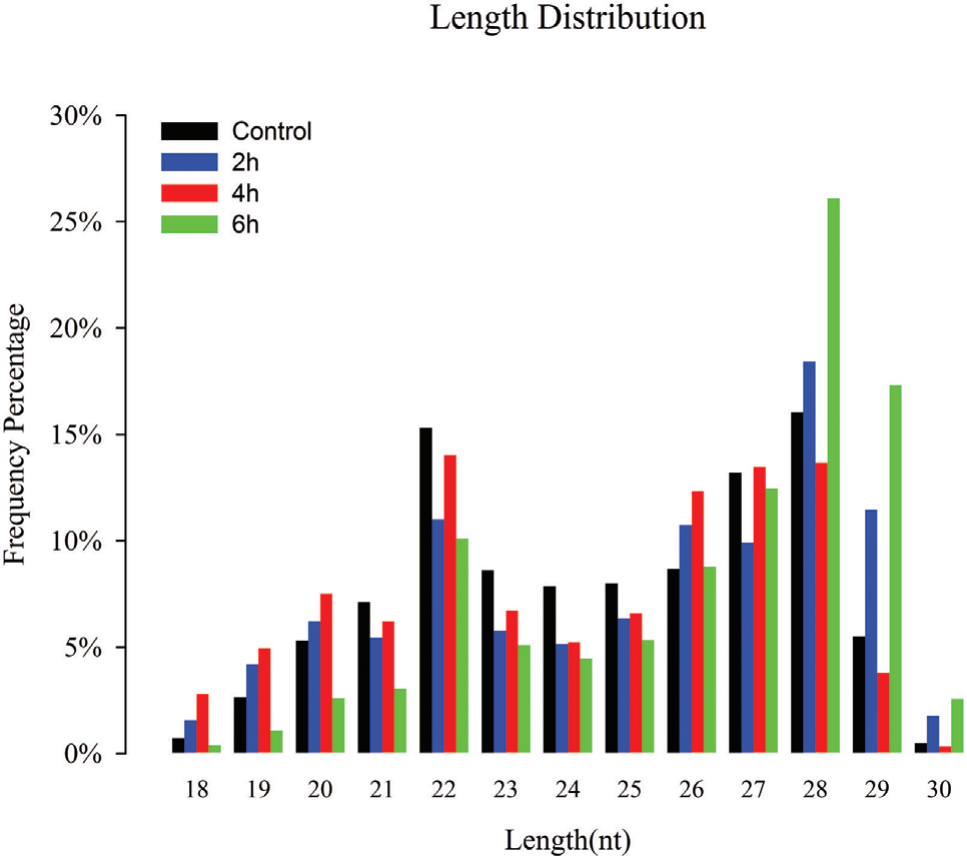
Size distribution of destruxin A-responsive small RNAs (sRNAs) in the libraries of *Plutella xylostella*. Different colors represent different libraries. *x*-Axis represents sRNA length distribution and *y*-axis represents frequency percentage.

Four sRNA libraries were constructed for control, 2, 4, and 6 h, and 11,586,075, 15,391,118, 12,893,039, and 13,876,345 high-quality reads were obtained, respectively. The low-quality sequences, reads with 5′ contaminants and without a 3′ primer or insert tag, and reads shorter than 18 nt, were eliminated; subsequently, 11,492,082 (99.19%), 15,048,896 (97.78%), 12,519,758 (97.1%), and 13,777,285 (99.29%) clean reads in the control, 2, 4, and 6 h were obtained for further analysis, respectively (Table S1 in Supplementary Material). The sRNA length distribution of the four libraries exhibited that most of the sRNAs ranged from 18 to 30 nt with 26 to 28 nt sRNAs being most abundant following 22 and 29 nt (Figure 1). Among the total and unique clean reads, 21,944,702 and 39,83,00 sRNAs between control and 2 h; 20,268,898 and 3,22,472 sRNAs between control and 4 h; 20,334,789 and 3,80,546 sRNAs between 6 h and control; 23,648,245 and 4,36,969 sRNAs between 2 and 4 h; 24,227,618 and 5,33,391 sRNAs between 6 and 2 h; and 22,256,011 and 4,35,747 sRNAs were common between 6 and 4 h, respectively (Figure S1 in Supplementary Material).

### Genome Mapping and sRNA Annotation

Of the clean reads, 7,359,318, 8,346,568, 6,685,514, and 7,855,270 reads from control, 2, 4, and 6 h accounted for 64.04, 55.46, 53.4, and 57.02%, respectively, and were mapped to the genome of *P. xylostella* (GCA_000330985.1) (Table S2 in Supplementary Material). The annotation of sRNAs was carried out by following priority rule of rRNA, etc.; (GenBank > Rfam) > known miRNA > repeat > exon > intron (38). The clean reads were categorized into miRNA, rRNA, snRNA, snoRNA, tRNA, and unannotated. The composition of the sRNA classes in each library is displayed in Figure S2 in Supplementary Material.

### Identification of Destruxin A-Responsive Known miRNAs

**Figure 2:**
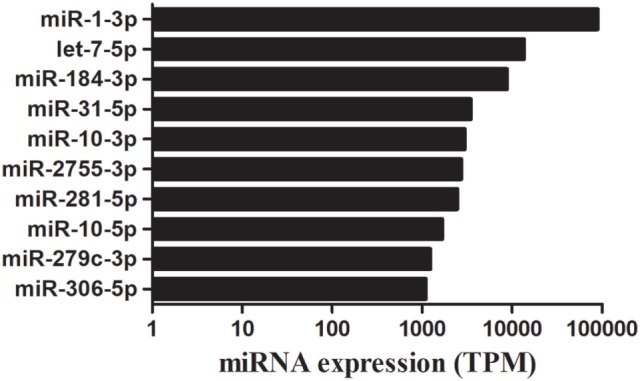
A time course of destruxin A-responsive top 10 abundantly expressed microRNA (miRNAs) in *Plutella xylostella*. Average expression value of all time courses is presented here. TPM, transcripts per million.

The bioinformatic analysis was carried out to identify destruxin A-responsive known miRNAs in *P. xylostella*. Although miRNAs are among the most intensively studied molecules since last two decades, deciding what is and what is not a miRNA has been difficult and it has been argued that miRBase is riddled with false positives (sequences that are not derived from bona fide miRNA genes) (39–41). Recently, Etebari and Asgari (42) re-annotated miRNAs of *P. xylostella* and after removing previously reported low confidence precursor miRNAs (pre-miRNAs), finally, they confirmed 114 highly confident pre-miRNAs of *P. xylostella* following strict criteria. Therefore, in the present study, after successful mapping of clean reads against *P. xylostella* genome, the mapped miRNA sequences were matched to miRNAs reported by Etebari and Asgari (42). Our analysis initially identified, based on sequence similarity, in total, 187 mature miRNAs. Then, precursor sequences of these mature miRNAs were aligned to those reported by Etebari and Asgari (42), and 99 highly confident pre-miRNAs were confirmed, which produced 167 of 187 mature miRNAs. Our analysis indicated that pre-miRNA sequences of the remaining 20 conserved miRNAs were not detectable in the current assembly of *P. xylostella* genome. To obtain more reliable results, we removed those known miRNAs with read count < 10 in all libraries, and, finally, remaining 124 known miRNAs with precursor sequences (Table S3 in Supplementary Material), and 17 miRNAs without precursor sequences (Table S4 in Supplementary Material) were retained for further analysis. The remaining sequences that were not matched to conserved miRNAs were used to predict novel miRNAs by using the miRDeep2 program. The most abundant miRNA was pxy-miR-1-3p following pxy-miR-184-3p, pxy-let7-5p, and pxy-miR-31-5p. The top 10 most highly expressed miRNAs in the four libraries of *P. xylostella* are presented in Figure 2.

### Identification of Destruxin A-Responsive Novel miRNAs

The novel miRNAs with predicted precursor and secondary structures were identified by using miRDeep2 software (43), and, in total, 44 potential novel miRNAs from all the libraries were identified (Table S5 in Supplementary Material) following the standard criteria of novel miRNA prediction with miRDeep score > 1, randfold *P-*value < 0.05, and MFE < -19 kcal/mol. The most abundant miRNA was pxy-novel-mir-1 following pxy-novel-mir-38, and pxy-novel-mir-3 (Table S5 in Supplementary Material).

### Identification of Destruxin A-Responsive Differentially Expressed miRNAs

**Figure 3:**
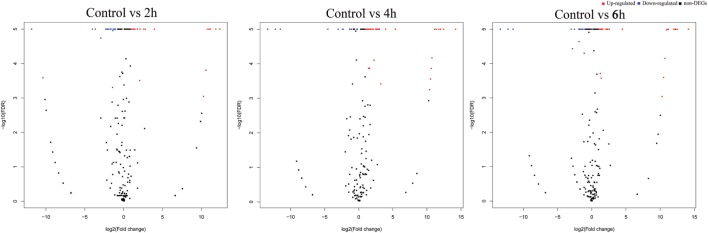
Volcano plots of destruxin A-responsive differentially expressed microRNA (miRNAs) in *Plutella xylostella*. The volcano plots represent differentially expressed miRNAs at different time courses (2, 4, and 6 h) compared to control.

The differential expression analysis of known miRNAs among the treatments exhibited that 26, 53, and 24 miRNAs were identified as differentially expressed between control and 2, 4, and 6 h, respectively (Figure 3). Of these, 12, 48, and 18 miRNAs were upregulated while 14, 5, and 6 miRNAs were downregulated, respectively (Table S6 in Supplementary Material).

The novel miRNA differential expression analysis indicated that 16, 17, and 18 miRNAs were identified as differentially expressed between control and 2, 4, and 6 h, respectively (Figure 3). Of these, 10, 12, and 11 miRNAs were upregulated whereas 6, 5, and 7 were downregulated, respectively (Table S7 in Supplementary Material). Overall, most of the differentially expressed miRNAs were upregulated in all the treatments.

### Target Prediction of Destruxin A-Responsive Known and Novel miRNAs

**Figure 4:**
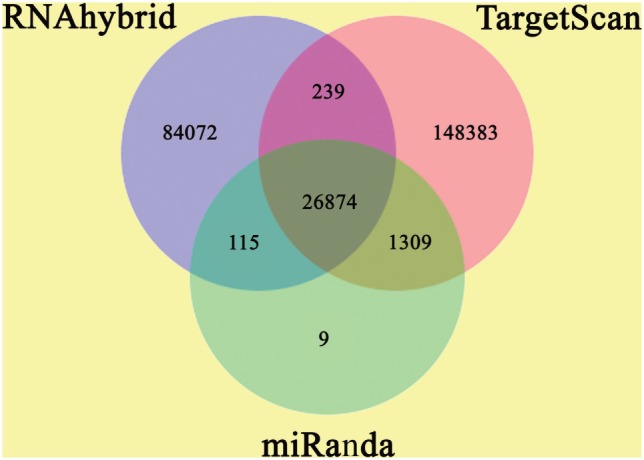
Target prediction of potential target genes of destruxin A-responsive microRNAs (miRNAs) in all libraries of *Plutella xylostella*. Venn diagram shows the number of miRNA targets and their overlapping spots predicted by the three programs (RNAhybrid, miRanda, and TargetScan).

The annotation of known and novel miRNA targets is necessary for defining their roles in response to destruxin A treatment. All the annotated genes of *P. xylostella* were screened using three different algorithms (RNAhybrid, miRanda, and TargetScan) to predict the potential binding sites for destruxin A-responsive miRNAs of *P. xylostella*. Our target prediction results identified 27,073 common spots between RNAhybrid and TargetScan, 26,989 between RNAhybrid and miRanda, and 28,183 between TargetScan and miRanda. When the target prediction results of all three software were combined, 26,874 common spots were detected and selected for further analysis (Figure 4).

### GO Enrichment and KEGG Pathway Analysis of Destruxin A-Responsive miRNA Target Genes

**Figure 5:**
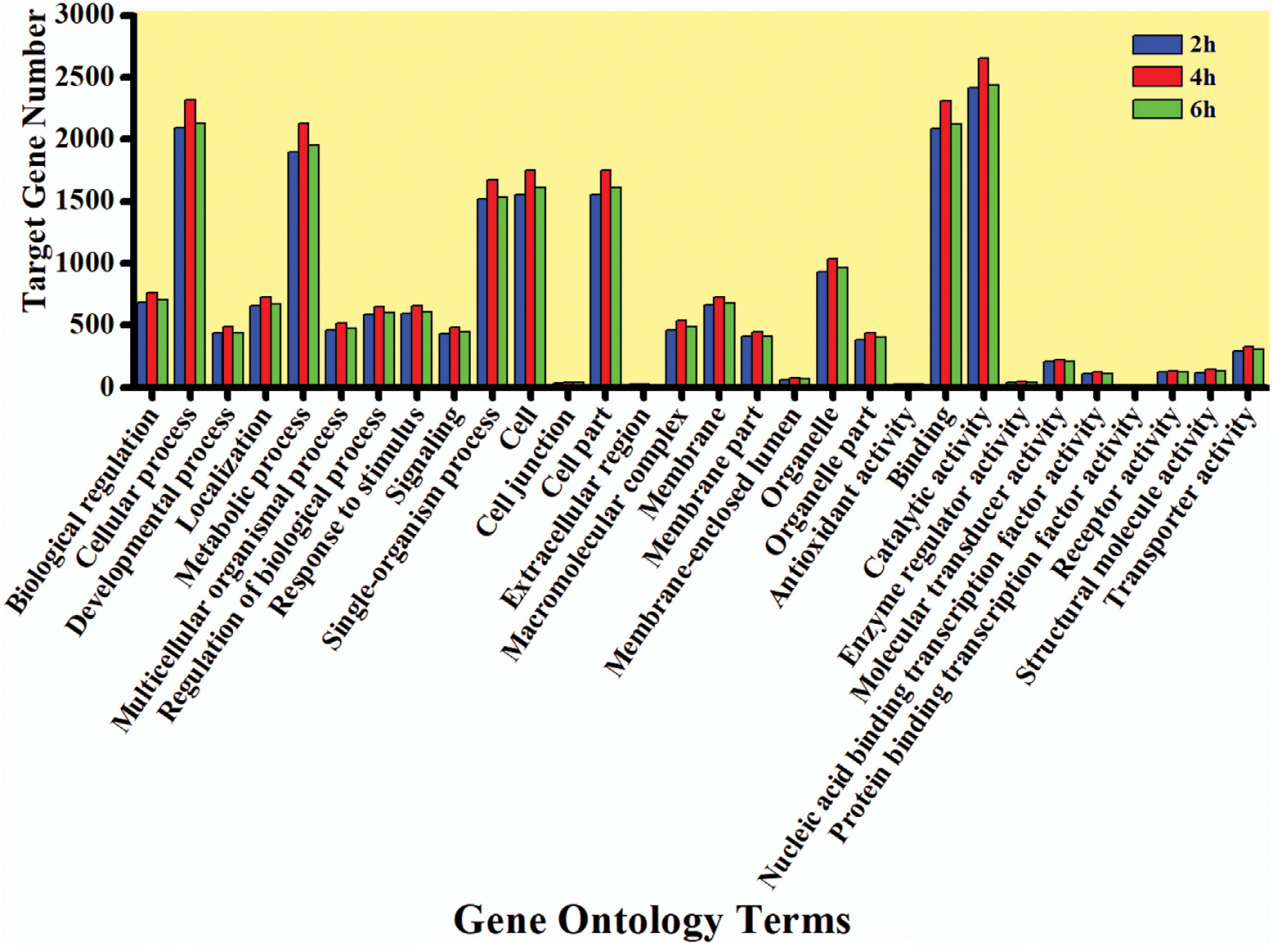
Gene ontology (GO) annotation of target genes of destruxin A-responsive microRNA in all libraries of *Plutella xylostella*. The abscissa is the GO annotation and the ordinate left is the gene number.

**Figure 6:**
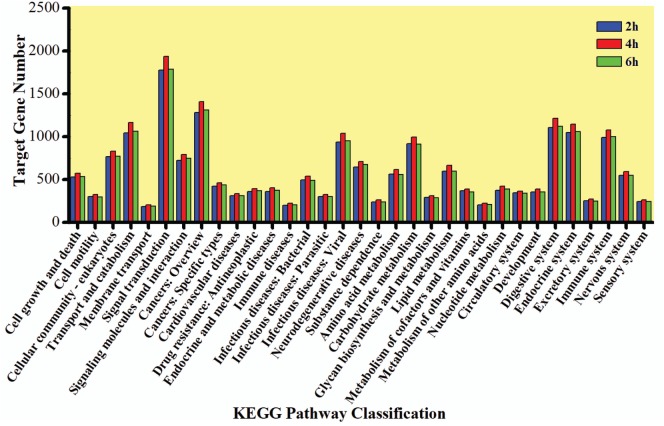
Kyoto Encyclopedia of Genes and Genomes (KEGG) classification of target genes of destruxin A-responsive microRNAs in all libraries of *Plutella xylostella*. The abscissa is the KEGG classification and the ordinate left is the gene number.

The GO enrichment analysis was carried out to gain knowledge of the potential function of each putative target gene. The GO enrichment analysis exhibited that cellular process and metabolic process, cell and cell part, and catalytic activity and binding were the most enriched categories in the biological process, cellular component, and molecular function, respectively (Figure 5). The KEGG classification system categorized destruxin A-responsive miRNA target genes into different groups. In the gene repertoire of 2, 4, and 6 h, the top five enriched groups among KEGG categories included signal transduction, cancers, digestive system, immune system, and transport and catabolism (Figure 6).

**Figure 7:**
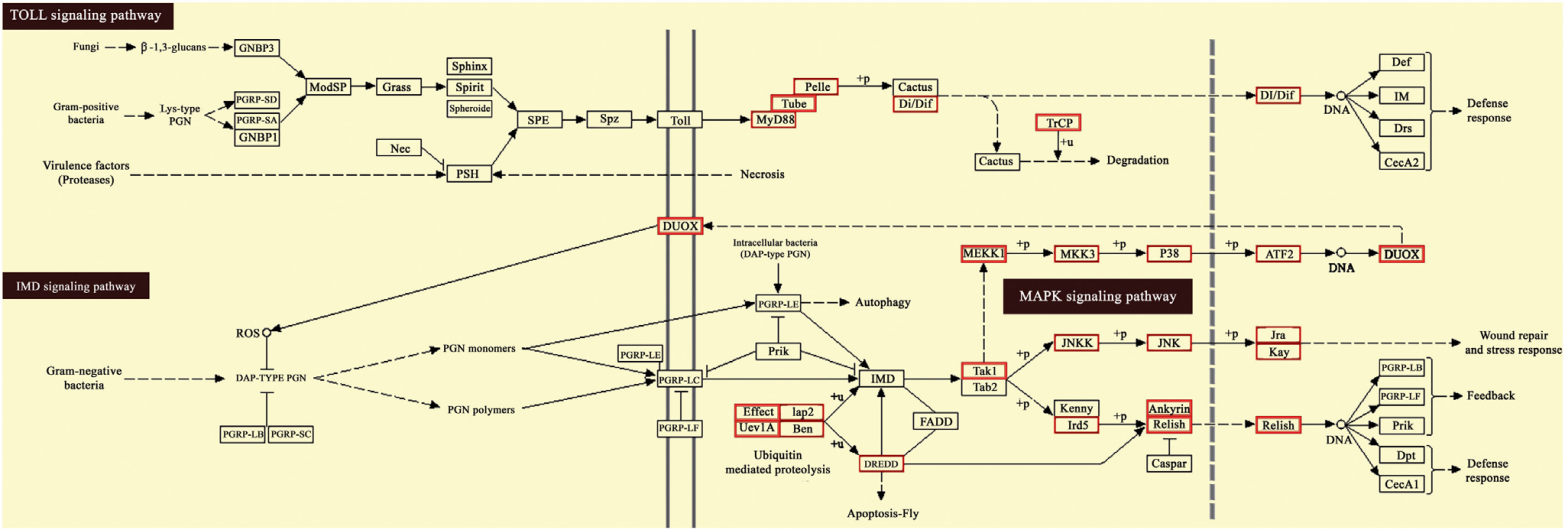
Kyoto Encyclopedia of Genes and Genomes (KEGG) pathway map for Toll and IMD signaling pathway. The red boxes indicate target genes of destruxin A-responsive microRNAs (miRNAs) in the particular pathways.

Moreover, KEGG pathway analysis of predicted target genes was carried out to identify potential pathways regulated by destruxin A-responsive differentially expressed miRNAs. The results exhibited that target genes were enriched in cell adhesion and focal adhesion (Figures S3 and S4 in Supplementary Material), as well as several immunity-related signaling pathways, including toll signaling pathway, IMD signaling pathway, and JAK–STAT signaling pathway (Figure 7 and Figure S5 in Supplementary Material).

### Validation of Differentially Expressed miRNAs by RT-qPCR

**Figure 8:**
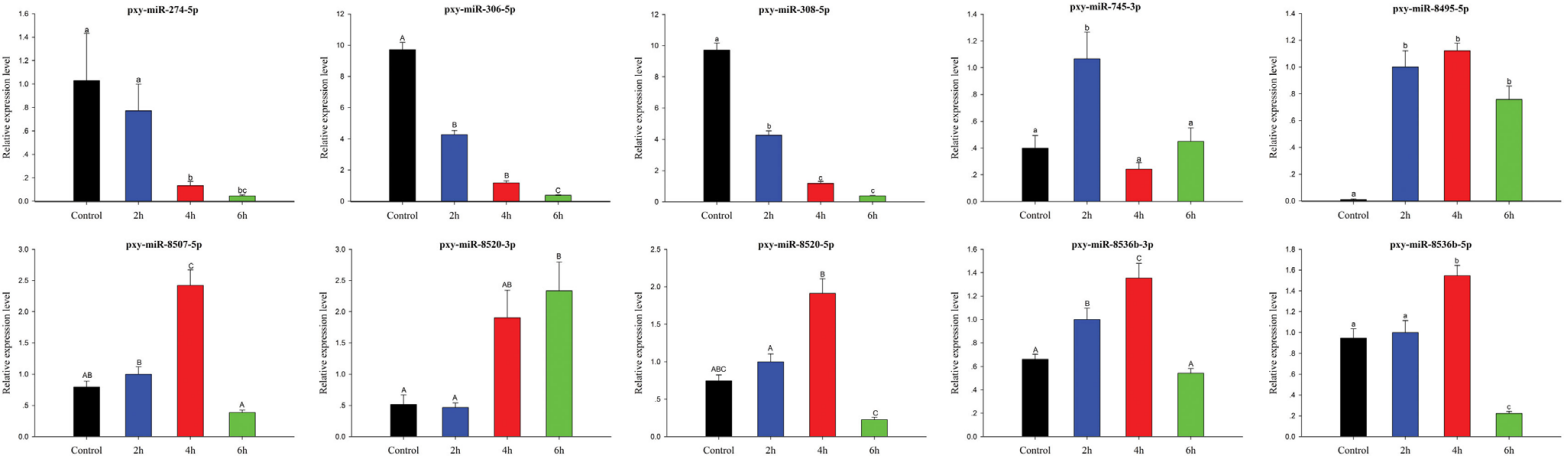
Expression of significantly differentially expressed microRNA (miRNAs) at different time courses after destruxin A injection. Each vertical bar represents the mean ± SEM (*n* = 3) for various time courses. Statistically significant differences in different groups are indicated by different letters (lowercase letters at *P* < 0.05 and capital case letters at *P* < 0.01).

To validate sRNA sequencing results, 10 randomly selected differentially expressed miRNAs among control, 2, 4, and 6 h were analyzed by RT-qPCR (Figure 8). The results exhibited that the trend of expression level for the selected miRNAs showed a little discrepancy to that of RNA-Seq analysis, which might be due to the differences in the sensitivity, specificity, and algorithm between the two techniques.

## Discussion

The diamondback moth, *P. xylostella*, has become the major lepidopteran pest of *Brassica* owing to its strong ability of resistance development to a wide range of insecticides. In the present scenario, there is a need to develop novel biological control methods, to reduce harmful effects of insecticides, as alternative control (44). The entomopathogenic fungi such as *M. anisopliae, B. bassiana*, and *Isaria fumosorosea* have gained an increased attention for controlling insect pests as they are considered to offer an environmentally friendly alternative to insecticides (45). The secondary metabolites of fungi, such as destruxins, are reported to have substantial insecticidal activity (46–48) and can destroy the immune system of insects (16, 23). miRNAs, the key regulators of gene expression at the posttranscriptional level, play a pivotal role in host–pathogen interaction. In recent years, the number of miRNAs isolated from insect species is increasing as new ones are deposited in the databases. Although the role of miRNAs in insect development is well understood, there is limited information available about the role of miRNAs in insect–pathogen interaction. Therefore, the present study aimed to acquire the immunity-related miRNAs in *P. xylostella* treated with destruxin A at three-time courses (2, 4, and 6 h). For this purpose, four sRNA libraries were constructed and sequenced using the high-throughput Illumina sequencing resulting in a total of 187 known and 44 novel miRNAs identified in the four libraries.

The sRNAs are classified into miRNAs, small interfering RNAs, and piwi-interacting RNAs (piRNAs) according to their size (49). Our length distribution results showed two peaks; one at 22 nt and the second at 28 nt, indicating to typical miRNAs and piRNAs. The piRNAs are commonly found in sRNA libraries of insects (50–52) and function as sequence-specific silencers in many organisms (53).

Some conserved miRNAs, such as miR-1, let-7, miR-10, and miR-306, showed abundant expression in the four libraries indicating that these miRNAs play vital regulatory roles in *P. xylostella*. These miRNAs were also discovered to be abundantly expressed in sRNA libraries of other insects (52, 54); however, a low copy number of miR-1, a conserved miRNA, was detected after parasitization in a previous report (55). Moreover, it is worth mentioning that the expression levels of miR-2755, miR-184, and miR-281 were also abundant in all the treatments indicating that these miRNAs might play a crucial role in *P. xylostella* immunity against microorganisms.

The differential expression analysis indicated that in total, 26, 53, and 24 known miRNAs were differentially expressed after treatment of *P. xylostella* with destruxin A at 2, 4, and 6 h, respectively. It is worth mentioning, of these, some conserved miRNAs such as miR-263, miR-279, miR-306, miR-2a, and miR-308 also showed differential expression in the present study. Previous studies reported that these miRNAs (miR-263, miR-279, miR-306, miR-2a, and miR-308) were also differentially expressed after treatment with pathogens indicating that these miRNAs play important role in the regulation of immunity-related genes in insects (52, 55, 56). Notably, miR-263 has been shown to regulate immunity-related signal transduction in *Galleria mellonella* by affecting the gene expression of tumor necrosis factor receptor superfamily (56). In addition, miR-263 was hypothesized to be involved in the regulation of signal modulation in *Manduca sexta* by affecting the gene expression of serine protease inhibitors (52). However, in the present study, a lower expression level, less than onefold, was observed. Similar to our findings, miR-279 was also upregulated when *P. xylostella* was parasitized with *Diadegma semiclausum* indicating that it plays an important role in the immune response of *P. xylostella* against parasitoids and microorganisms (55). Interestingly, pxy-miR-306 not only showed differential expression after treatment with destruxin A but was also listed in the top 10 highly expressed miRNAs in our study. It suggests that miR-306 family might be involved in the regulation of immunity-related genes. Previously, it has been found that miR-306 family is associated with Cry1Ab resistance in *Ostrinia furnacallis* (57). Of note, pxy-miR-308 was commonly differentially expressed in all the treatments indicating that it might play a vital role in immunity of *P. xylostella* against destruxin A by regulating immunity-related genes. According to a previous report, Mse-miR-308 was supposed to be involved in extracellular signal transduction and melanization (52).

To better understand the function of each putative target gene, GO annotation and KEGG pathway analysis were performed. According to GO annotation, the differentially expressed target genes were mainly classified in a cellular process, cell, catalytic activity, and metabolic process (Figure 5). Previously, similar GO annotation of differentially expressed target genes was obtained in *O. furnacallis* in response to *Bacillus thuringiensis* and *Wolbachia*-responsive miRNAs in *Tetranychus urticae* (57, 58).

It is of interest that KEGG pathway annotation of target genes resulted in the identification of several immunity-related signaling pathways, including toll signaling pathway, IMD signaling pathway, JAK–STAT signaling pathway, and cell adhesion molecules pathway. The toll signaling pathway is a crucial pathway in the innate immunity (59), and in insects, it is responsible for fungi and Gram-positive bacteria recognition (60). The presence of miRNA target genes in toll signaling pathway in the current study suggests that some miRNAs regulate innate immunity against the entomopathogenic fungi *via* toll pathway. Previous studies on the model insect *D. melanogaster* have also indicated the role of toll pathway in immunity against fungi and pointed out that toll-mutant flies were highly susceptible to fungi (61).

Interestingly, the cell adhesion molecules pathway was highly enriched in the current study. Cell adhesion molecules, glycoproteins, are expressed on the surface of a cell and are reported to play a vital role in biological processes, including immune response (62). These cell adhesion molecules are categorized as integrin family, selectins, immunoglobulin superfamily, and cadherins. The immunoglobulin superfamily proteins are reported to play an important role in facilitating specific interactions with particular pathogens (63). Taken together, the current study clearly presents patterns of differentially expressed miRNAs in *P. xylostella* treated with destruxin A at different time courses.

## Conclusion

Concluding our findings, the present study adopted high-throughput sRNA sequencing to systematically screen out destruxin A-responsive immunity-related miRNAs in *P. xylostella*. According to our information, this is the first study about immunity-related miRNA profiles of *P. xylostella* in response to pathogens especially on secondary metabolites of entomopathogenic fungi like destruxin A. In the current study, several miRNAs that may regulate immunity through their targets and related pathways were identified. Among them, miR-263, miR-279, miR-306, miR-2a, and miR-308 are worthy to mention as these showed differential expression at different time courses and have also been predicted to regulate immunity in the other insects. Our findings will provide a strong foundation for further functional studies of miRNAs regulating immunity-related target genes of *P. xylostella* in response to microorganisms.

## Ethics Statement

Our work conﬁrms to the legal requirements of the country in which it was carried out.

## Author Contributions

Conceived and designed the experiments: FJ, MS, and XiaoxX. Performed the experiments: MS, JX, and XiaoxX. Analyzed the data: MS, XiaojX, JY, XZ, and JX. Contributed reagents/materials/analysis tools: SL and QH. Wrote the manuscript: MS and XiaoxX. Revised the manuscript: FJ and YX.

## Conflict of Interest Statement

The authors declare that the research was conducted in the absence of any commercial or financial relationships that could be construed as a potential conflict of interest.
