# Supplementary material for: Genome-Wide Identification of Destruxin A-Responsive Immunity-Related MicroRNAs in Diamondback Moth, Plutella xylostella
Source: Front Immunol. 2018 Feb 8;9:185. doi: 10.3389/fimmu.2018.00185 (PMC5809476; doi:10.3389/fimmu.2018.00185)
Supplement: Supplementary file 8 [file Data_Sheet_1.docx]

**Supplementary Material Figure 1. The common and specific sRNAs of *Plutella xylostella* at different time points.**

(A) summary of total sRNAs between ck and 2 h (B) summary of unique sRNAs between ck and 2 h (C) summary of total sRNAs between ck and 4 h (D) summary of unique sRNAs between ck and 4 h (E) summary of total sRNAs between 6 h and ck (F) summary of unique sRNAs between 6 h and ck (G) summary of total sRNAs between 2 h and 4 h (H) summary of unique sRNAs between 2 h and 4 h (I) summary of total sRNAs between 2 h and 4 h (J) summary of unique sRNAs between 2 h and 4 h (K) summary of total sRNAs between 6 h and 2 h (L) summary of unique sRNAs between 6 h and 2 h (M) summary of total sRNAs between 6 h and 4 h (N) summary of unique sRNAs between 6 h and 4 h.

**Supplementary Material Figure 2. Small RNA composition of ck, 2 h, 4 h, and 6 h in *P. xylostella***

**Supplementary Material Figure 3. KEGG pathway map for Cell adhesion molecules**

The red boxes indicate target genes of microRNAs in the particular pathways.

**Supplementary Material Figure 4. KEGG pathway map for Focal adhesion**

The red boxes indicate target genes of microRNAs in the particular pathways.

**Supplementary Material Figure 5. KEGG pathway map for Jak-STAT signaling pathway**

The red boxes indicate target genes of microRNAs in the particular pathways

**Supplementary information Table 1.** The classification of destruxin A-responsive total small RNAs of the *Plutella xylostella* by sequencing

**Supplementary information Table 2.** The mapping statistics of destruxin A-responsive small RNAs from four libraries of *Plutella xylostella*

**Supplementary information Table 3**. Expression profiles of destruxin A-responsive known miRNAs with their precursor sequences in *Plutella xylostella*

**Supplementary information Table 4.**  Expression profiles of destruxin A-responsive known miRNAs whose precursor sequences were not found in *Plutella xylostella* genome

**Supplementary information Table 5.** Expression profiles of destruxin A-responsive novel miRNAs with their precursor sequences in *Plutella xylostella*

**Supplementary information Table 6.** Differential expression of destruxin-A responsive known miRNAs between control and 2 h, 4 h, and 6 h in *Plutella xylostella*

**Supplementary information Table 7.** Differential expression of destruxin-A responsive novel miRNAs between control and 2 h, 4 h, and 6 h in *Plutella xylostella*
