# Supplementary figures and images for: Genome-Wide Identification of Destruxin A-Responsive Immunity-Related MicroRNAs in Diamondback Moth, Plutella xylostella
Source: Front Immunol. 2018 Feb 8;9:185. doi: 10.3389/fimmu.2018.00185 (PMC5809476; doi:10.3389/fimmu.2018.00185)

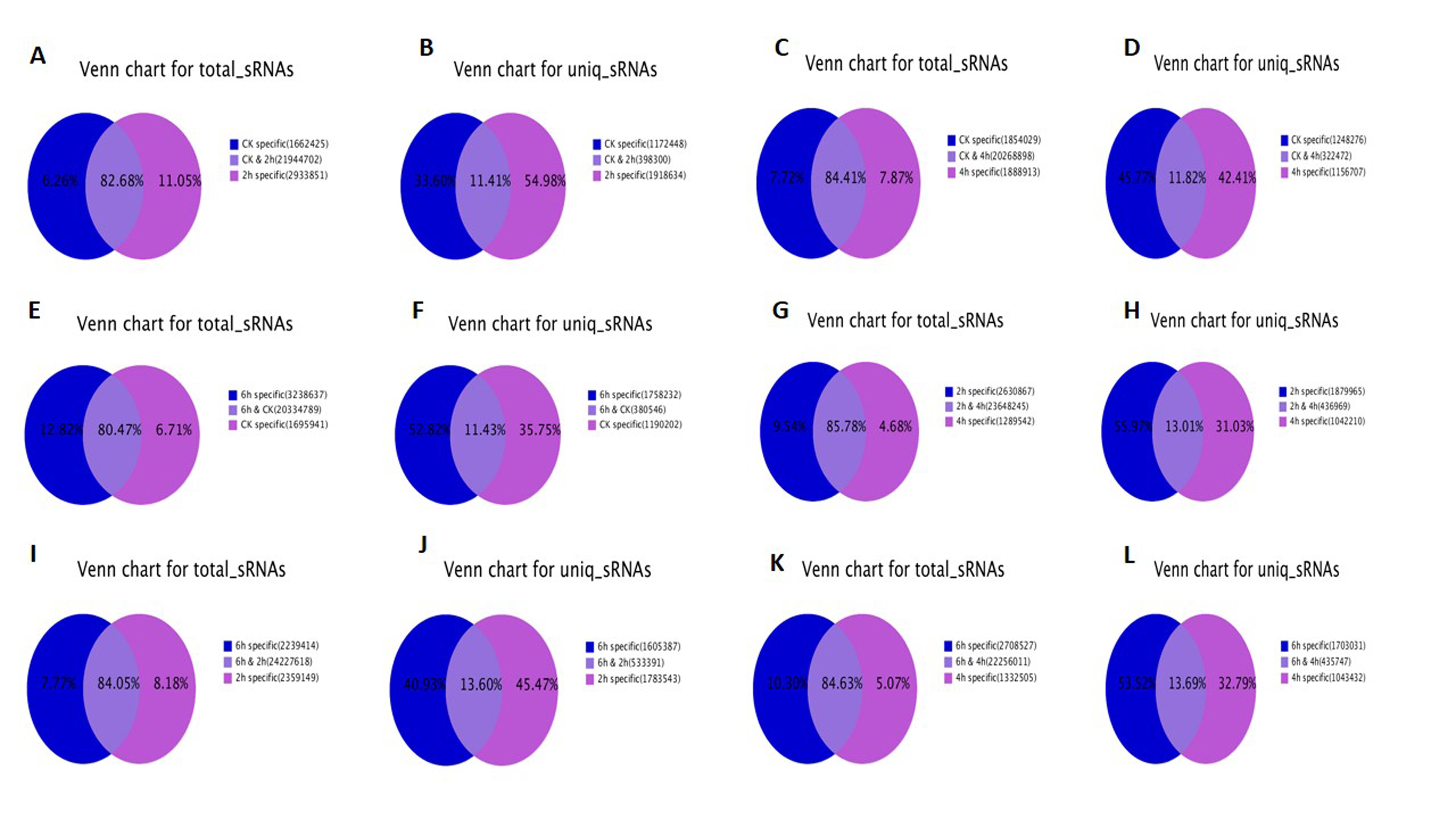

Supplement: Supplementary file 9 [file Image_1.tif]

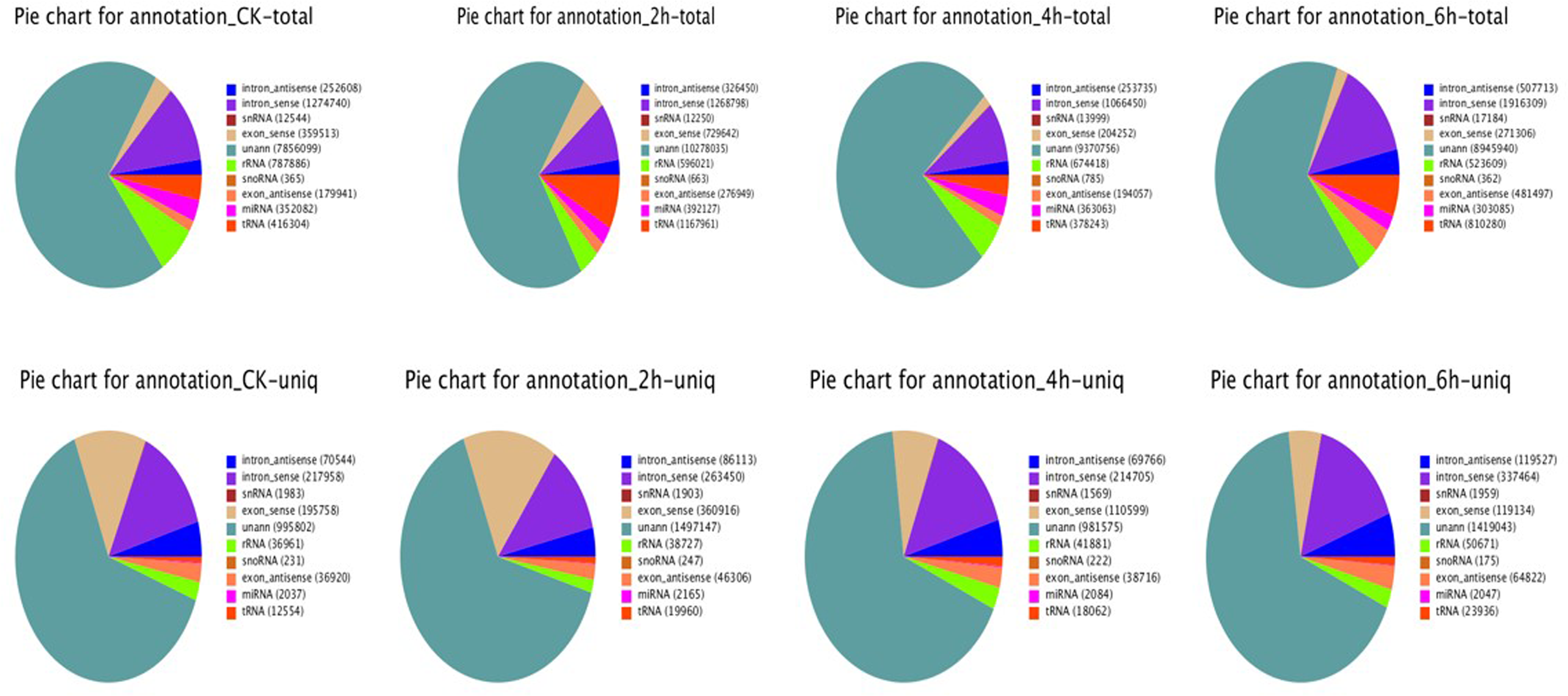

Supplement: Supplementary file 10 [file Image_2.tif]

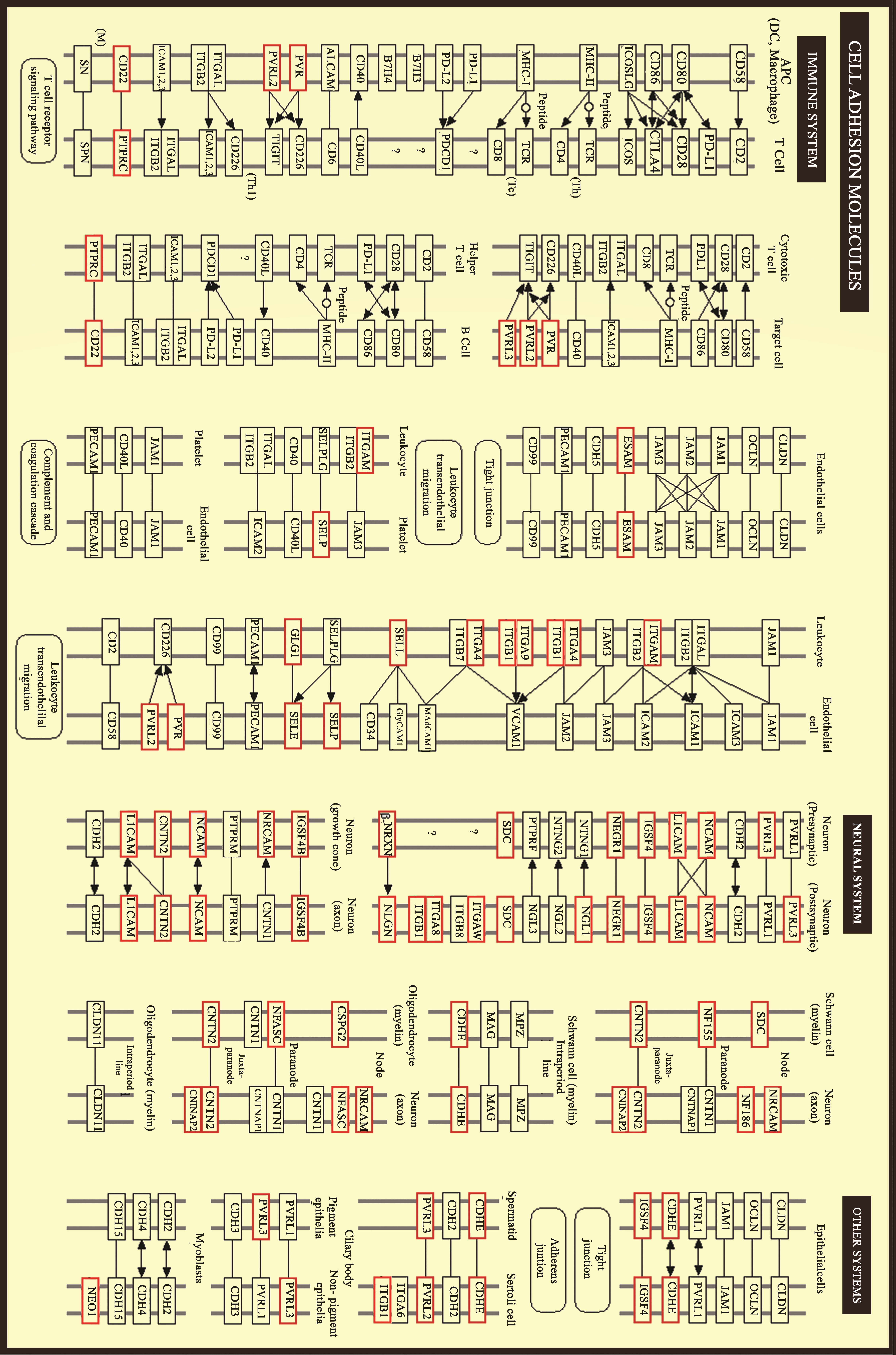

Supplement: Supplementary file 11 [file Image_3.jpeg]

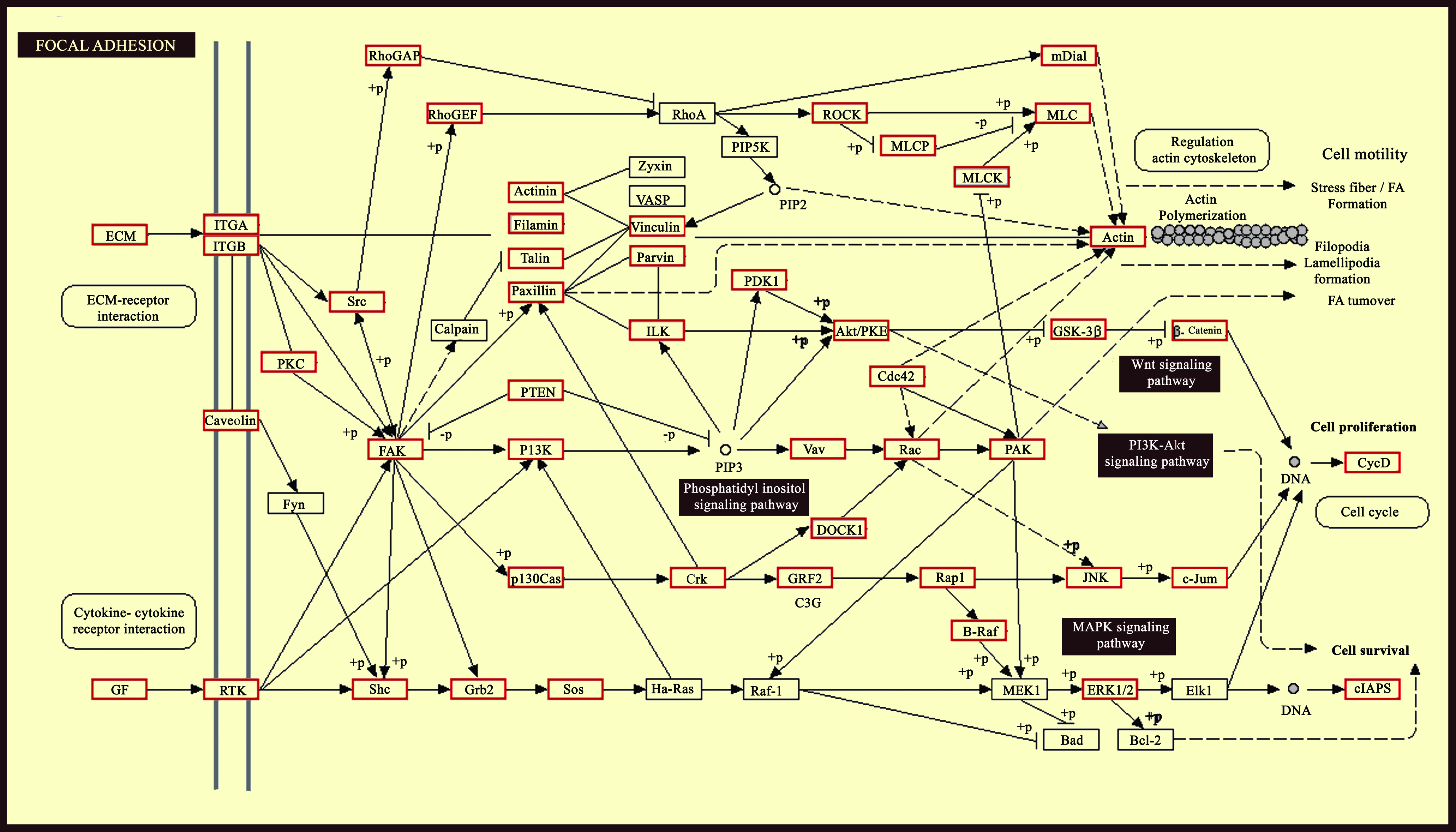

Supplement: Supplementary file 12 [file Image_4.jpeg]

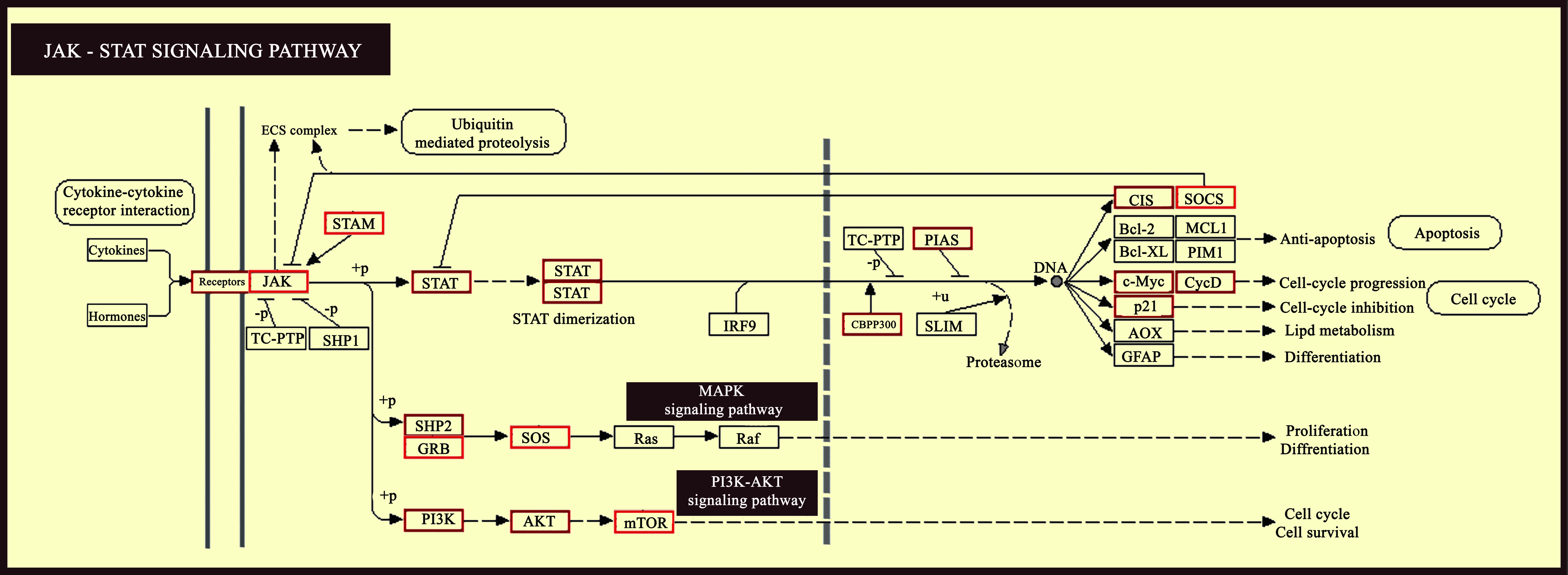

Supplement: Supplementary file 13 [file Image_5.jpeg]
